# Personalizing Care for Informal Heart Failure Caregivers: Challenges and Practical Implications

**DOI:** 10.1007/s11897-025-00703-2

**Published:** 2025-04-08

**Authors:** Emma Säfström, Maria Liljeroos

**Affiliations:** 1 Department of Health, Medicine and Caring Sciences, Linköping University, Linköping, Sweden; 2 Centre for Clinical Research Sörmland, Uppsala University, Eskilstuna, Sweden; 3 Department of Medicine, Mälarsjukhuset hospital, Eskilstuna, Sweden

**Keywords:** Informal caregiver, Heart failure, Person-centered care, Intervention

## Abstract

**Purpose of Review:**

To summarize articles describing how to personalize care for heart failure (HF) informal caregivers on the basis of the literature review results. We also describe informal caregivers’ preferences and wishes regarding personalized care.

**Recent Findings:**

Recent interventions to support informal caregivers were delivered face-to-face or online in group or individual sessions. The sessions embraced various elements, including coaching on setting personalized goals and developing problem-solving strategies. The interventions improved a range of variables, such as caregiver burden, quality of life, depression, stress and anxiety. Informal caregivers described personalized care as being in a partnership, clear communication and coordination of care.

**Summary:**

Several intervention studies reported positive caregiver effects; however, they were small, and sometimes, the interventions were only briefly described. A deeper and more comprehensive understanding of the experiences and needs of informal caregivers is essential before new tailored interventions can be developed.

**Supplementary Information:**

The online version contains supplementary material available at 10.1007/s11897-025-00703-2.

## Introduction

Informal caregivers to patients living with heart failure (HF) play an important role in supporting patients’ well-being and self-care management. An informal caregiver can be a relative, a neighbor or a close friend. The results from the European Social Survey 2017 revealed that, on average, 34% of the population in 20 European countries described themselves as informal caregivers due to long-term illness, disability or problems related to old age. Approximately 8% provided care for a minimum of 11 h per week [1]. Owing to this burdensome situation, informal caregivers of patients with HF are at risk for negative caregiver outcomes such as poor psychological well-being, poorer health, burden, and lower quality of life (QOL). The growing prevalence of home-based care places even greater demands on informal caregivers [2, 3].

Informal caregivers describe numerous unmet needs, such as a lack of information and knowledge on how to support patients and how to stay healthy themselves. Furthermore, healthcare professionals do not always recognize the caregiver’s burdensome situation. The support provided has been described as insufficient, and caregivers often find themselves “left in the dark” and miss genuine interactions with healthcare professionals [4]. Younger adult caregivers expressed difficulties in receiving appropriate information and support. Instead, they actively search out information relevant to them and their family via alternative networks. For example, they use social media to access peer support and information that fits their situation [5].

There is a relatively small body of research exploring how to support HF dyads and an even smaller body of research exploring what support informal caregivers need to preserve health and QOL. This is despite the evidence that patients and informal caregivers influence each other’s health in important ways [6].

The person-centered nursing framework has been recognized to operationalize person-centeredness in nursing practice. The framework has four key components: nursing prerequisites, the care environment, the person-centered nursing process and person-centered nursing outcomes. The person-centered nursing process centers on activities that operationalize person-centered nursing and especially focus on the patient and others significant to them. Central aspects include working with persons’ beliefs and values, sharing decision-making, engaging authentically, providing holistic care and being sympathetically present. As the person-centered nursing framework suggests, nursing interventions should include informal caregivers and not be limited to the patient. The expected outcome of the framework is a good care experience [7, 8].

As chronic conditions increase, the contributions of informal caregivers are likely to increase. Nurses face challenges in how to include caregivers in the strategic planning and operational stages of care tasks and in supporting them in their caregiving roles. Therefore, the aim of this study was to summarize articles describing how to personalize care to informal HF caregivers on the basis of the results of the literature review. Additionally, we describe informal caregivers’ preferences and wishes regarding personalized care.

## Method

### Design

We conducted an integrative review and followed standard reporting guidelines [9]. The sequential process included identifying the research question, identifying and selecting relevant studies, charting the data and comprehensively summarizing the results [10].

### Literature Search

The search was performed in March 2025 in the EBSCO database. The following search string was utilized:

((MH Heart failure+) OR (TI (“heart failure” OR “cardiac failure” OR “myocardial failure” OR ”heart insufficiency”) OR AB (“heart failure” OR “cardiac failure” OR “myocardial failure” OR ”heart insufficiency”))) AND (((MH (Palliative care OR Family nursing OR Rehabilitation OR Health education OR Education OR Teaching OR Counseling) AND (MH Caregivers)).

#### OR

(TI (“palliative care” OR rehabilitation OR nursing OR “family-centered care” OR “family-focused care” OR “experimental studies” OR support* OR educat* OR teach* OR advis* OR advice* OR counsel* OR intervention* OR therap* OR program* OR burden OR psycho* OR “future plan*” OR coping OR “problem solving” OR advocate* OR “day care” OR daycare) N10 (family OR families OR parent* OR friend* OR relative* OR spouse* OR partner* OR husband* OR wife OR wives OR child OR children OR “close person*” OR “significant other*” OR caregiv* OR “care giver*” OR caretak* OR carer* OR partner* OR sibling* OR neighbour* OR “informal care” OR dyad*))

#### OR

(AB (“palliative care” OR rehabilitation OR nursing OR “family-centered care” OR “family-focused care” OR “experimental studies” OR support* OR educat* OR teach* OR advis* OR advice* OR counsel* OR intervention* OR therap* OR program* OR burden OR psycho* OR “future plan*” OR coping OR “problem solving” OR advocate* OR “day care” OR daycare) N10 (family OR families OR parent* OR friend* OR relative* OR spouse* OR partner* OR husband* OR wife OR wives OR child OR children OR “close person*” OR “significant other*” OR caregiv* OR “care giver*” OR caretak* OR carer* OR partner* OR sibling* OR neighbour* OR “informal care” OR dyad*)))

#### AND

(YR 2018–2025)

### Selection and Analysis of Studies

In total, 1935 articles were identified in the database. For inclusion in this analysis, studies were required to be published or in press between 2018 and March 2025, peer reviewed and published in the English language. Literature reviews, protocols, and case studies were excluded.

**Fig. 1:**
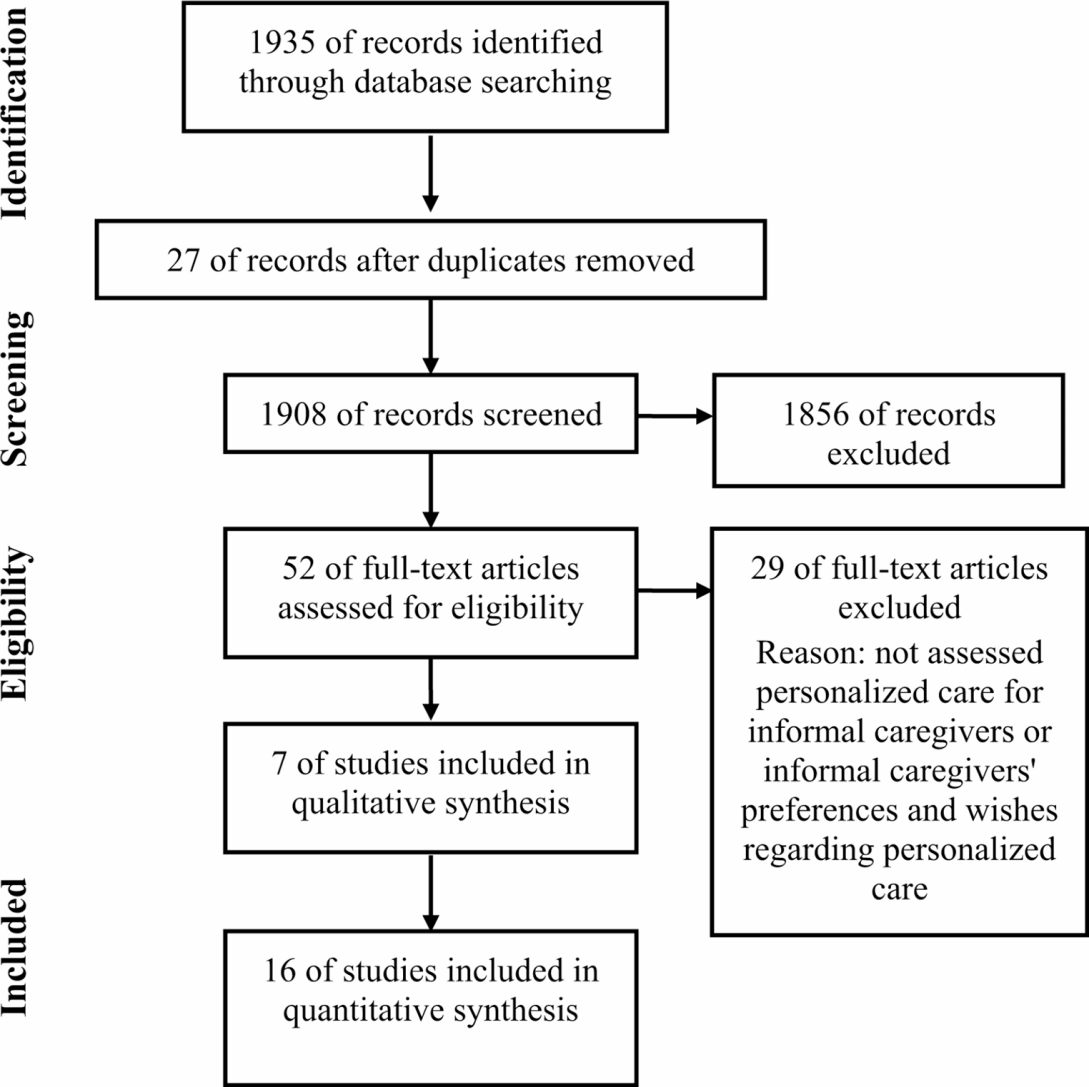
Prism flow diagram of the study selection process

First, we excluded duplicates (*n* = 27). Among the 1908 remaining articles, 1856 were excluded from the analysis for various reasons (Fig. 1). 52 articles were read in full-text and 29 were excluded since they did not assess personalized care for informal caregivers, Supplementary material. 23 articles were included in the analysis. All the references from the included publications were traced to identify other potentially relevant research.

To strengthen the content validity, both authors independently screened the titles and abstracts of the retrieved studies, and the full texts of potentially relevant articles were further read for inclusion. Discussion of disagreements preceded the final decision on inclusion.

The quality of the included articles was assessed using the Mixed Methods Appraisal Tool (MMAT) [11]. The MMAT is specifically designed for reviews incorporating both qualitative and quantitative methods. There is no scoring system for quality based on the MMAT; instead, we report the quality appraisal as either high or low, along with a brief explanation for any low scores based on the MMAT questions.

Data extraction was conducted independently by the authors on a predefined extraction table, which included (1) general information about the studies (first author, publication date, and country); (2) aim of the study; (3) method; (4) sample size; (5) personalized content of the intervention; and (3) results.

The quantitative and qualitative articles were analyzed descriptively separately to answer the aims of the review. Finally, we synthesized the results by applying the person-centered nursing framework [8].

## Results

### Study Characteristics

This study included 23 articles in total, including both quantitative (*n* = 16) and qualitative (*n* = 7) studies (Table 1). The quantitative articles describe personalized interventions that have been evaluated, including informal caregivers, and the qualitative articles describe their experiences and preferences for personalized care.

**Table 1:** Description of the journal articles included in the systematic review

Author, year, country	Aim of the study	Stated method	Sample size/sex	Personalized content of the intervention	Results	Quality appraisal
**Included in the quantitative analysis**						
Abshire Saylor, et al. 2023a. United States	To 1) test the feasibility and acceptability of the Caregiver Support program and 2) provide preliminary pilot data on the effects of Caregiver Support on caregiver quality of life, burden, and fatigue. In addition, we examined evidence for self-efficacy as a possible mediator of improvement.	Randomized waitlist control design.	38 caregivers 92% female	Nurse-led, online individual sessions. Components in the sessions addressed holistic caregiver assessment, life purpose, action planning, resources, and future planning. 1) discussing the caregiver’s life holistically to identify strengths and unmet needs, 2) exploring values and developing a life purpose statement, 3) mapping social support to involve others in caregiving, 4) making connections to community and palliative care resources, and 5) building a sustainability plan for addressing future needs. Every visit was concluded with action planning to address self-care and caregiving needs.	Improvements with large effect sizes (.52–.88) were seen in mental quality of life, self-efficacy, and social support. Improvement with a moderate effect size was noted for caregiver burden.	High (Fulfills all criteria in MMAT except 2.4)
Abshire Saylor, et al. 2023b, United States	To describe action plans, action plan achievement and life purpose statements of HF caregivers in the Caregiver Support pilot randomized waitlist control trial.	Descriptive	22 caregivers 91% female	Nurse-led, online individual sessions. Components in the sessions addressed holistic caregiver assessment, life purpose, action planning, resources, and future planning. 1) holistic assessment of caregiving needs; 2) a discussion of caregiving in the context of the caregiver’s life purpose to provide a rationale for action planning; 3) co-development of short-term action plans to address the caregiver’s self-care needs; 4) exploration of social, community, and palliative care resources to support personal and caregiving needs; and 5) creation of a sustainable action plan for addressing any future needs. Every visit was concluded with action planning to address self-care and caregiving needs.	Action plans comprised five categories: personal health and well-being, social support, home environment, instrumental support and other. The most common topics of life purpose statements were faith and self-care/actualization. A total of 67% of the action plans were achieved.	High
Alaei et al 2024, Iran	To determine the effectiveness of the supportive-educational program, based on the COPE (creativity, optimism, planning and expert advice) care model, on the quality of life and caregiver burden of family caregivers of HF patients.	A single-blind study, two-group, three-stage clinical trial	90 caregivers 69% female	Face-to-face as well as digital sessions. Activities included determining and prioritizing problems and education on applying the problem-solving process based on the COPE model.	The caregivers in the intervention group reported significantly improved quality of life (p < 0.05) and caregiver burden (p < 0.05) immediately and three months after the intervention compared to the control group.	High
Bandini et al, 2021, United States	To evaluate the feasibility and acceptability of disease-specific group visits for patients with HF and their caregivers.	Feasibility trial	21 caregivers 80% females 36 patients 50% females	Facilitator-led, face-to-face group sessions that focused on active care planning The primary focus was to help participants think about and clarify their health goals, values, and preferences. The video-based PREPARE for Your Care was accompanied by a workbook with values clarification exercises and culturally relevant discussion questions.	Participants felt comfortable discussing active care planning in groups. They understood the information covered and could identify and clarify their healthcare values. Being in a group and listening to a diverse range of perspectives facilitated the process of identifying and clarifying preferences.	High
Borji et al 2019, Iran	To determine the effect of spiritual care training on reducing anxiety in family caregivers of patients with HF in Ilam, Iran	Semi-experimental study	71 caregivers 69% female	Research-led face-to-face sessions. Discussing the role of patience, altruism, forgiveness, recitation and praying.	There was a significant difference between the control group and the intervention group in reported anxiety after three weeks (p < 0.001).	Low (Does not fulfill criteria 2.1, 2.4 and 2.3 is not stated)
Dionne-Odom et al 2020, United Kingdom	To test the effect of ENABLE CHF-PC compared with usual care.	A 2-site, single-blind, randomized clinical trial	158 caregivers 85% female	Nurse-led, individual phone sessions. The sessions included discussions on problem-solving, the role of values, how to ask for help, and identifying and building a support team. They were guided by the Charting Your Course-caregiver guidebook.	Caregiver quality of life, mood and reported caregiver burden were evaluated. No significant differences were found between the control and intervention groups.	Low Does not fulfill criteria 2.3, 2.4 and 2.5)
Hwang et al. 2022, South Korea	To evaluate the potential of an intervention designed to reduce stress using CBT among caregivers of patients with HF. The specific aims of this study were to examine the effects of an 8-week CBT intervention compared to an attention control condition on physiological (i.e., salivary cortisol) and psychological outcomes in HF caregivers at postintervention and 6-month follow-up and to describe effect sizes.	A pilot randomized controlled trial	30 caregivers 54% female 30 patients 54% female	Face-to-face individual cognitive behavioral therapy (CBT) sessions led by a researcher who was trained in CBT	Caregivers in the intervention group had significant within-group improvements in perceived stress (*p* =.011), stress symptoms (*p* =.017), depression (*p* =.002), and anxiety (*p* =.006) from baseline to post-intervention. The control group only had a significant within-group change in anxiety (*p* =.03).	High
Irani et al. 2021, United States	To evaluate the feasibility, acceptability, and preliminary efficacy of an eHealth dyadic teamwork intervention compared to an attention control condition.	Two-arm randomized, pilot clinical trial	28 caregivers 79% females 28 Patients 64% males	Interactive sessions, called the eSMART-HF intervention. The following topics were covered: setting shared goals, communicating timely, accurately, and effectively, managing emotions, enhancing mutual respect and assessing progress. Patients and caregivers had access to separate sessions.	A large effect size (0.85) was identified for caregivers’ mental quality of life. No p-values are reported.	High (Fulfills all criteria in MMAT except 2.4)
Lyon et al 2025, United States	To (1) determine the feasibility and acceptability of the TCU program and (2) explore preliminary change in physical and mental health and dyadic management	Feasibility and acceptability study with a two-arm randomized controlled trial with pretest–post-test design	37 caregivers 78% female	Trained social-work students led couples in online or telephone sessions called Taking Care of Us (TCU). The session was communication-based and relationship-focused, designed to support the couple in reflecting on their strengths and areas of challenge, and tailored for their needs.	Almost all of the participants in TCU reported that the topics were relevant to them and that their relationship had improved. For partners, a medium effect size was found for physical health (d = 0.74) at the post-test and at 5 months (*d* = 0.73). There was also a medium effect for care strain at 5 months (*d* = − 0.53). There were no significant effects for depressive symptoms (*d* = − 0.15) or anxiety (*d* = − 0.10).	High Fulfills all criteria in the MMAT except 2.5
Marzban et al 2024, Iran	To evaluate the effect of emotional freedom techniques (EFTs) on anxiety and caregiver burden of family caregivers of patients with HF.	Quasi-experimental	91 caregivers 67% female	Researcher-led individual face-to-face sessions applying emotional freedom techniques (EFT). The training sessions included identifying anxiety factors, negative experiences and emotions, and factors increasing caregiver burden. Tapping started focusing on the factor(s) mentioned and continued until the caregiver gave the lowest possible score to her/his anxiety	A significant difference (p < 0.001) between groups regarding mean scores of anxiety and caregiver burden were found immediately and one month after the intervention	High (Fulfills all criteria in the MMAT except 2.4)
Piamjariyakul et al. 2024, United States	To test whether a home palliative care intervention (FamPALcare) would improve family caregiver and patient HF-related health status and their depression/anxiety scores at 3- and 6-month endpoints. Another purpose was to verify the feasibility, fidelity, helpfulness and costs of FamPALcare remote telephone intervention delivery.	Randomized controlled trial (RCT)	39 caregivers 77% females 39 patients 67% males	Nurse-led telephone coaching sessions. Sessions addressed caregiver involvement in homecare and aimed to identify each family’s HF home care needs. They included coaching on palliative symptom management, strategies for discussing end-of-life, and guidance on advanced directives.	Caregivers in the intervention group had significantly lower scores on caregiving burden and depression after three months compared to the control group.	Low (Does not fulfill 2.2. 2.4 and 2.5 is not stated)
Pucciarelli et al 2024, Italy	To evaluate the effectiveness of MI on mutuality in HF patient– caregiver dyads.	Secondary outcome analysis of the MOTIVATE-HF randomized controlled trial,	Caregivers (n = not clearly stated) 75% female Patients (n = not clearly stated) 58% male	Nurse-led, face-to-face MI sessions and three telephone follow-up contacts. The sessions were individual or dyadic, depending on the study arm. The MI focused on supporting self-efficacy, developing problem-solving abilities, respecting patients' and caregivers' choices, and avoiding contrasts.	Among caregivers, the changes in mutuality scores significantly increased after nine months in arm 2 (MI for patients and caregivers) compared with arm 3 (standard care; difference, 0.2; 95% confidence interval [CI], 0.0–0.3; P =.0314).	High
Purcell et al. 2023, Scotland	The research questions were: (1) How do ‘real-world’ patient and caregiver outcomes and REACH-HF costs compare with those in the randomized trial? and (2) What are the service-level facilitators and barriers to REACH-HF implementation?	Mixed-method, single-arm, pre-post design study	56 caregivers 76% female 136 patients 72% male	Health professional-facilitated face-to-face and telephone contacts. A “family and friends’ resource” to increase caregivers' understanding of HF to enable them to support the person with HF’s self-care and wellbeing.	Caregiver contribution to self-care domains of symptom perception and management improved significantly.	Low (The quantitative and qualitative components and are not integrated)
Thodi et al. 2023, Greece	To examine the effect of a nurse-led educational intervention delivered through either a combined home/telephone sessions or telephone-only sessions in recently hospitalized HF patients and their caregivers on outcomes of patients and caregivers. The effect on caregiver burden, feelings of guilt and health-related quality of life.	Single-center, single-blind, randomized, parallel-controlled study	57 caregivers 84% female 57 patients 84% males	Nurse-led face-to-face and by-telephone dyadic educational sessions. The content included information on managing one's health and well-being, getting needed help, and problem-solving strategies.	After six months, the caregivers in the intervention group significantly improved in health-related quality of life, caregiver burden and caregivers’ feelings of guilt compared to the control group.	High
Yang et al. 2024, China	To assess the efficacy of the family FOCUS program.	Two-arm parallel group randomized control trial	71 caregivers 61% females 71 patients 61% males	Researcher-led online sessions for each dyad. They set personalized self-management goals and got a family-specific plan following each family’s traits.	Caregiver contributions to self-care maintenance, self-care management, and symptom perception improved significantly.	High
Wingham et al. 2019, United Kingdom,	To compare the caregiver outcomes between the REACH-HF and control groups; and to report the views and perceptions of caregivers on their experience of using the REACH-HF intervention.	Multicenter randomized controlled trial	97 caregivers 78% females	Nurse-led, face-to-face and by telephone. ‘Family and friends’ resource’– for caregivers, including advice on providing support, becoming a caregiver, managing the caregiver’s health and well-being and getting help.	Caregivers in the intervention group reported significantly higher scores in Caregivers’ Contribution to Self-care of HF Index than caregivers in the control group after 12 months. Qualitative results showed that caregivers in the intervention group made positive changes in how they supported their partner.	High (Fulfills all criteria in the MMAT but 2.5 is not described)
**Included in the qualitative analysis**						
Alleman et al, 2019, Sweden	To explore the perceptions of ICT solutions as supportive aids among family members of persons with HF.	Focus group interviews with ICGs.	23 family members of persons affected by HF, 18 women	NA	ICGs described multiple uses for information and communications technology (ICT) and agreed that ICT could provide access to relevant sources of information from which family members could potentially exchange support. ICT was also considered to have its limitations and was out of scope for some but with expected use in the future.	High
Blanck et al, 2021, Sweden	Clarify the meaning of support given and received by informal carers to relatives with chronic obstructive pulmonary disease or HF.	Individual interviews with ICG.	14 interviews with 12 informants, all females	NA	A comprehensive understanding of the meaning of support is twofold: it is a self-evident struggle for the good life of their relatives, and they want to be carers in partnership.	High
Fitzsimons et al, 2019, Ireland	To explore caregivers" experience when caring for a loved one with advanced HF at the end of life and to identify any unmet psychosocial needs.	Semi-structured, one-to-one interviews with current and bereaved caregivers.	30 interviews included 20 current ICGs and 10 bereaved ICGs, 15 females	NA	Caregivers in advanced HF need clearer communication regarding the diagnosis and prognosis of their loved one’s condition to help with the uncertainty of their situation. More coordinated service provision is required to address physical and emotional challenges from diagnosis through bereavement.	High
Robinson et al, 2024, New Zealand	To explore family caregivers’ experiences of accessing information from healthcare professionals when caring for someone with HF.	A qualitative exploratory study design was adopted using a critical realist approach.	A total of 15 family caregivers participated, 5 females.	NA	A thematic analysis identified three themes related to accessing information from healthcare professionals: 1) gaining access to healthcare professionals 2) developing, understanding and translating information and 3) receiving information in a timely manner.	High
Sampaio et al, 2019, Portugal	To explore the meaning of being a family caregiver for a relative with advanced HF in their own home	Phenomenological- hermeneutical method. ICGs for relatives with advanced HF participated in two reflective interviews over a four-month period.	10 ICGs, all females	NA	Findings support that family caregivers require participation in the planning and execution of their relative’s health care.	High
Schutz SE, Walthall HE, 2022, United Kingdom	To explore the needs and experiences of caregivers caring for a person with HF through a qualitative interview approach.	ICG were interviewed using a semi-structured approach, and the data was analyzed using thematic analysis.	17 informants. No demographic or biographical data were collected.	NA	Caregiving can negatively impact ICGs' health and well-being and involves complex care delivery for which they receive little support. ICGs report having significant information needs to understand the reasons for the care they provide yet feel marginalized by health care professionals.	High
Östman et al, 2019, Sweden	To describe continuity of care as perceived ICGs who care for patients with HF.	Semi-structured interviews.	Ten women and five men	NA	ICGs perceive continuity of care when they have access to care and treatment and when ICGs collaborate, regardless of whether healthcare is provided by primary care, municipalities or specialist clinics.	High

HF; Heart failure

ICG; Informal Caregivers

### Personalized Interventions Delivered To Informal HF Caregivers

#### Study Designs

Most of the quantitative articles reported findings from randomized control trials [12–22]; however, this review also included Quasi experimental [23, 24], pre-post design [25] and feasibility studies [26, 27].

#### Personalized Content in Interventions

The personalized content included a range of different interventions, such as assessment of informal caregiver needs [18]; coaching to set personalized self-management goals [13]; problem-solving strategies [15, 17, 19]; development of family-specific plans [13]; coaching on how to ask for help and build a support team [17]; discussions and exercises to clarify goals, preferences and values [26]; enabling dyadic teamwork [20, 27]; and education on palliative HF symptoms [14].

The interventions also omitted a strengths-building support program [12] that included action planning [18]. Some interventions include psychotherapeutic interventions such as cognitive behavioral therapy (CBT) [16], emotional freedom techniques (EFTs) [23] and motivational interviewing (MI) [21]. One article reported a spiritual intervention that included sessions on altruism, forgiveness, recitation and praying [24].

#### Types of Sessions

Most interventions were applied as individual sessions [12, 16–18, 23], however three studies had dyadic sessions [13, 21, 27]. We found only one article where the sessions were performed as group sessions [26]. Some articles did not explicitly state whether the sessions were individual or in a group [15, 19, 20, 24].

The interventions were delivered digitally or by phone [12, 13, 17, 18, 20, 27] but also face-to-face [15, 16, 21, 23, 24] or in combination with face-to-face and digitally/by phone [19, 22, 25]. Nurses often led the sessions [12, 15, 17, 18, 21, 22]. Nevertheless, the sessions were also led by researchers [13, 23] or social work students [27]. In some articles, it was not clearly stated [19, 20, 24].

#### Intervention Outcomes

The interventions were found to have a significant effect on caregiver burden [14, 15, 19, 23, 27], caregiver quality of life [15, 19], perceived stress [16], caregiver anxiety [16, 23, 24], caregiver depression [14, 16], caregiver feelings of guilt [15], caregiver contribution to heart failure self-care [13, 22, 25] and mutuality scores [21] and increased confidence in the caregiver role over time [22].

### Informal Caregiver’s Experiences with and Preferences for Personalized Care

A total of seven qualitative studies reported on informal caregivers’ preferences and wishes regarding personalized care.

Personalized care for informal caregivers was described as being in a partnership with the patient with HF and healthcare professionals. To achieve this goal, caregivers wish to be treated as persons, be met with respect, be taken seriously, be listened to and receive adequate help when needed. Informal caregivers also require participation in planning and execution of the care and positive feedback from nurses was perceived as empowering [28].

Furthermore, information and support should be tailored and timely [29–31]. The busyness of nurses left some family feeling unable to ask for the information they were seeking and lack of consistency with the information provided by nurses added to a sense of uncertainty for some participants [31].

Caregivers in advanced HFs request clearer communication regarding the diagnosis and prognosis of their loved one’s condition to help with the uncertainty of their situation [32].

If the quality and quantity of information presented to carers are not adequate, it fails to facilitate caregivers’ understanding of the clinical issues their loved one is facing, including symptoms, treatment options, and supportive needs. A lack of information negates the possibility of shared decision-making for patients and their caregivers [32]. Improving service provision and coordination between different disciplines is also needed. Healthcare professionals need to collaborate, regardless of whether care is provided by primary care, municipalities or specialist clinics, to achieve continuity of care and seamless transitions, which has also been emphasized as an important cornerstone in personalized care [32, 33].

Family members describe multiple uses for information and communications technology (ICT) and agree that ICT could provide access to relevant sources of information from which family members can potentially exchange support [34].

### Application of the person-centered Nursing Framework To Personalize Care for HF Informal Caregivers

The person-centered process, a component of the person-centered nursing framework, includes the patient and others significant to them. Table 2 presents an application of the person-centered process to informal HF caregivers.

**Table 2:** Application of the person-centered process supporting informal HF caregivers

Central aspects of the Person-Centered Nursing Processes	Activities performed by nurses that operationalize personalized care for informal caregivers
Working with beliefs and values	- Explores values and preferences - Explores health goals - Mapps social support
Sharing decision-making	- Supports problem-solving - Informs on how to manage one’s health and well-being - Coaches on how to handle palliative symptom management - Supports the creation of an Action plan
Engaging authentically	- Keeps close contact also with the informal caregiver - Provides clear, tailored and timely information and support - Provides written contact information to the nurse - Helps navigate the healthcare system to provide seamless care - If possible, use ICT to exchange support to the informal caregiver
Providing holistic nursing care	- Offers spiritual care - Discuss the caregiver’s life holistically - Discuss the role of praying when appropriate - Identifies the whole family’s needs - Creates a family-specific plan adapted to each family’s trait - Assesses caregiver burden
Being sympathetically present	- Forms a partnership with the patient and informal caregiver - Proactively bring up the emotional aspect and offer emotional support - Recognizes the caregiver as a person and acknowledges the caregiver’s emotions - Identifies the caregiver’s perceptions about their situation

ICT; information and communications technology

## Discussion

This literature review summarized publications describing interventions tested to personalize care for HF informal caregivers. We also included qualitative publications describing caregiver experiences and personalized care preferences. To help nurses operationalize person-centeredness in practice, we have applied the findings and based further discussion on central aspects of the person-centered nursing framework [7, 8].

One problem in selecting articles to include in the review was the lack of a clear definition of personalized care concerning informal caregivers, and none of the included articles explicitly claim to be a personalized intervention. Therefore, on the basis of the person-centered nursing framework, we chose to include articles where the intervention was not strictly a standardized protocol; i.e., some parts of the intervention were adapted to the informal caregiver’s situation or personal needs.

### Working with Beliefs and Values

The person-centered framework emphasizes that nursing care should be provided to patients and others who are significant to them. This is also, to the greatest degree, applicable in the case of HF caregiving. A recent network analysis, including 355 HF dyads, revealed extensive interconnections among dyadic health components such as depression, anxiety, resilience, and perceived control. The findings highlight the importance of considering the dyad as a whole when providing care [35].

Few interventions used dyadic or group sessions where patients and informal caregivers could meet others in the same situation. This is problematic since previous studies have demonstrated that the HF patient and caregiver should be considered a dyadic whole. Moreover, informal caregivers prefer group sessions, as they find support when meeting with others in the same situation [35, 36].

### Sharing Decision-Making

Providing information is a core requirement for shared decision-making and participation [7, 8]. A lack of knowledge and misconceptions about HF is an important barrier for self-care management [37]. Some articles included in this review highlight the importance of nurses educating and training informal caregivers. It was found to increase their skills in managing their loved one’s health conditions, which, in turn, can increase mutuality and shared decision making between the dyad [21]. Education can be provided not only face-to-face but also via ICT [34]; however, the information should be tailored and timely [22, 29]. Furthermore, this corresponds well with findings from a recent study. In a structural equation model, health literacy was found to have a direct effect on HF caregivers’ health-related QoL [38].

### Engaging Authentically

A longitudinal study assessing patients’ and caregivers’ prognostic understanding of HF argues that regular and recurring communication is important to enhance prognostic understanding [39]. Both informal caregivers and patients ask for continuous guidance and easy access to the HF nurse. Coordinated care is needed during the whole illness trajectory, not just for a limited time after diagnosis or hospitalization [32, 36]. Using e-health services is becoming more common regardless of age as a way to exchange support and information [40, 41]. However, it is still an opportunity that is not fully utilized and needs to be more widely implemented in clinical care.

### Providing Holistic Care

Providing holistic care includes meeting the spiritual and psychosocial needs of the family [7, 8]. This is especially important, as it is known that the diagnosis can imply negative emotions, inability to continue with work, financial burden and changes in family roles [37].

As a recent review concluded, nurses should be aware of the importance of ongoing assessment and evaluation of caregivers’ needs and assist them in providing more information and formulating coping strategies as needed [4]. Additionally, a recent cross-sectional study that included 410 informal caregivers of patients with HF suggested that nurses should proactively assess caregiver burden and design personalized support plans [38].

### Being Sympathetically Present

Sympathetically present has been described as the core of all other person-centered processes. It is a process that reinforces the individuality of persons and encourages nurses to focus on developing their “ways of being” [42]. The findings of this literature review suggest that, for the nurse to be sympathetically present with informal HF caregivers, the nurse should recognize the caregiver as a person, form a partnership with the informal caregiver, and offer emotional support [29, 30]. Furthermore, structured conversations between nurses and families improve perceptions of reinforcement, feedback, decision-making capability and collaboration with nurses among patients with HF and their family members [43].

### Expected Outcomes

The most frequent outcomes measured in the current review were caregiver burden, depression or anxiety, i.e., the aim was to reduce informal caregivers’ burden and negative emotions [12, 14–17, 19, 20, 22–24]. Furthermore, studies have reported effects on caregiver contributions to self-care management and maintenance, as well as symptom perception and management, which improved significantly when family support programs were used [13, 25]. As described by the person-centered nursing framework, when care is delivered in a person-centered manner, it results in a good care experience. However, none of the included articles evaluated whether the caregivers had a good experience with care.

### Methodological Considerations

This study has several limitations. First, the articles included in this literature review were limited to English and focused on research published within the past five years, which may have excluded some relevant literature. Second, we used a broad search string after consulting experienced university librarians. The search identified many articles, many of which were eliminated after the first screening. It is possible that some relevant articles were not identified at this stage. However, both authors screened all the records, and any disagreements were resolved via discussion until a consensus was reached.

## Conclusion

In conclusion, we found a small number of publications that tested different types of personalized interventions for informal HF caregivers. Several of these studies reported positive caregiver effects. Considering that several studies are small, methods and interventions are different, and some interventions are only briefly described, considerable uncertainty remains regarding the specific intervention components that yield the greatest benefit for informal caregivers. A deeper and more comprehensive understanding of the experiences and needs of informal HF caregivers is essential before new tailored interventions can be developed.

### Future Research

To develop a deeper understanding of informal caregivers’ needs regarding personalized care, well-designed qualitative studies that can stand on their own and provide reliable qualitative data are needed. Future interventions need to evaluate caregiver outcomes, for example, care satisfaction, and not only burden, depression or patient-related outcomes. When new interventions are designed, we suggest using co-design, including informal caregivers, as they can provide important insights into the content needed to provide personalized care.

## References of Importance

- Important
  - Bandini et al. 2021, United States.
  - A feasibility study evaluating study material that aimed to support active care planning.
  - Piamjariyakul et al. 2024, United States.
  - This randomized control study evaluated a palliative care intervention and reported significant results regarding caregiver burden and depression after three months.

- Very important:
  - Irani et al. 2021, United States.
  - The intervention in this randomized control study covers most of the central aspects of the person-centered nursing process, such as setting goals, timely communication, and managing emotions. The intervention had a significant effect on caregivers’ mental quality of life.
  - Blanck et al., 2021, Sweden.
  - This study provides comprehensive insights into how to provide person-centered care for informal caregivers.

## Electronic Supplementary Material

Below is the link to the electronic supplementary material.

Supplementary Material 1

## Data Availability

No datasets were generated or analysed during the current study.
