# Supplementary material for: Personalizing Care for Informal Heart Failure Caregivers: Challenges and Practical Implications
Source: Curr Heart Fail Rep. 2025 Apr 8;22(1):14. doi: 10.1007/s11897-025-00703-2 (PMC11978547; doi:10.1007/s11897-025-00703-2)
Supplement: Supplementary file 1 — Supplementary Material 1 [file 11897_2025_703_MOESM1_ESM.docx]

**Supplementary material**

Excluded manuscripts after of full-text assess not addressed personalized care for informal heart failure caregivers or informal caregivers' preferences and wishes regarding personalized care.

1. Aamodt, I.T. *et al.* (2022) ‘Informal Caregivers’ Experiences with Performing Telemonitoring in Heart Failure Care at Home-A Qualitative Study’, *Healthcare (Basel, Switzerland)*, 10(7). doi:10.3390/healthcare10071237.

2. Albulushi, A. *et al.* (2024) ‘Digital health technologies in enhancing patient and caregiver engagement in heart failure management: Opportunities and challenges’, *International journal of cardiology*, 408, p. 132116. doi:10.1016/j.ijcard.2024.132116.

3. Alvariza, A. *et al.* (2018) ‘Family members’ experiences of integrated palliative advanced home and heart failure care: A qualitative study of the PREFER intervention’, *Palliative & supportive care*, 16(3), pp. 278–285. doi:10.1017/S1478951517000256.

4. Baik, D. *et al.* (2022) ‘Caregiving experiences of older family caregivers of persons with heart failure: A mixed methods study’, *Geriatric nursing (New York, N.Y.)*, 48, pp. 51–57. doi:10.1016/j.gerinurse.2022.08.015.

5. Bangerter, L.R., Griffin, J.M. and Dunlay, S.M. (2018) ‘Qualitative study of challenges of caring for a person with heart failure’, *Geriatric nursing (New York, N.Y.)*, 39(4), pp. 443–449. doi:10.1016/j.gerinurse.2017.12.017.

6. Barnes, S. and Whittingham, K. (2020) ‘Informal carers’ experiences of caring for a person with heart failure in a community setting’, *Health & social care in the community*, 28(3), pp. 883–890. doi:10.1111/hsc.12919.

7. Clements, L. *et al.* (2023) ‘Improvement in Heart Failure Self-Care and Patient Readmissions with Caregiver Education: A Randomized Controlled Trial’, *Western journal of nursing research*, 45(5), pp. 402–415. doi:10.1177/01939459221141296.

8. Durante, A. *et al.* (2022) ‘Informal caregivers of people with heart failure and resilience: A convergent mixed methods study’, *Journal of advanced nursing*, 78(1), pp. 264–275. doi:10.1111/jan.15078.

9. Durante, A. *et al.* (2023) ‘Burden among informal caregivers of individuals with heart failure: A mixed methods study’, *PloS one*, 18(11), p. e0292948. doi:10.1371/journal.pone.0292948.

10. Eghøj M, Zinckernagel L, Brinks TS, Kristensen ALS, Hviid SS, Tolstrup JS, Dalal HM, Taylor RS, Zwisler AO. Adapting an evidence-based, home cardiac rehabilitation programme for people with heart failure and their caregivers to the Danish context: DK:REACH-HF study. Eur J Cardiovasc Nurs. 2024 Oct 21;23(7):728-736. doi: 10.1093/eurjcn/zvae037. PMID: 38526240.

11. Hjelmfors, L. *et al.* (2018) ‘Using co-design to develop an intervention to improve communication about the heart failure trajectory and end-of-life care’, *BMC palliative care*, 17(1), p. 85. doi:10.1186/s12904-018-0340-2.

12. Li X, Zhang J, Li J, Fang W, Zhang X, Fan X. Determinants of preparedness in family caregivers of patients with heart failure. Eur J Cardiovasc Nurs. 2025 Jan 30;24(1):35-43. doi: 10.1093/eurjcn/zvae107. PMID: 39132774.

13. Locatelli, G. *et al.* (2022) ‘Effectiveness of Motivational Interviewing on contribution to self-care, self-efficacy, and preparedness in caregivers of patients with heart failure: a secondary outcome analysis of the MOTIVATE-HF randomized controlled trial’, *European journal of cardiovascular nursing*, 21(8), pp. 801–811. doi:10.1093/eurjcn/zvac013.

14. Locatelli, G. *et al.* (2023) ‘The Impact of an Intervention to Improve Caregiver Contribution to Heart Failure Self-care on Caregiver Anxiety, Depression, Quality of Life, and Sleep’, *The Journal of cardiovascular nursing* [Preprint]. doi:10.1097/JCN.0000000000000998.

15. Matus, A. *et al.* (2024) ‘Social Determinants of Health are Associated with Coping of Informal Caregivers of Adults with Heart Failure’, *Clinical nursing research*, 33(5), pp. 334–343. doi:10.1177/10547738231223790.

16. McIlfatrick, S. *et al.* (2018) ‘“The importance of planning for the future”: Burden and unmet needs of caregivers’ in advanced heart failure: A mixed methods study’, *Palliative medicine*, 32(4), pp. 881–890. doi:10.1177/0269216317743958.

17. Nguyen, D.D. *et al.* (2024) ‘Association of Patient Reported Outcomes With Caregiver Burden in Older Patients With Advanced Heart Failure: Insights From the SUSTAIN-IT Study’, *Circulation. Heart failure*, 17(7), p. e011705. doi:10.1161/CIRCHEARTFAILURE.124.011705.

18. Noonan, M.C. *et al.* (2024) ‘Caregiver presence in a home-based cardiac rehabilitation programme improves the health-related quality of life of patients with heart failure’, *European journal of cardiovascular nursing*, 23(1), pp. 90–94. doi:10.1093/eurjcn/zvad031.

19. Peng, Y. *et al.* (2023) ‘Resilience in Informal Caregivers of Patients with Heart Failure in China: Exploring Influencing Factors and Identifying the Paths’, *Psychology research and behavior management*, 16, pp. 1097–1107. doi:10.2147/PRBM.S405217.

20. Piamjariyakul U, Young S, Hendrickson AE, Navia RO, Wang K, Smith CE. Palliative Home Care Based on Clinically Relevant Scientific Measures: A Cross-Sectional Study. Am J Hosp Palliat Care. 2024 Nov 28:10499091241304728. doi: 10.1177/10499091241304728. Epub ahead of print. PMID: 39606853.

21. Østergaard, B. *et al.* (2021) ‘Effect of family nursing therapeutic conversations on patients with heart failure and their family members: Secondary outcomes of a randomised multicentre trial’, *Journal of clinical nursing*, 30(5–6), pp. 742–756. doi:10.1111/jocn.15603.

22. Santos, G.C. *et al.* (2023) ‘Feasibility, acceptability, and outcome responsiveness of the SYMPERHEART intervention to support symptom perception in persons with heart failure and their informal caregivers: a feasibility quasi-experimental study’, *Pilot and feasibility studies*, 9(1), p. 168. doi:10.1186/s40814-023-01390-3.

23. Sedlar, N., Lainscak, M. and Farkas, J. (2020) ‘Living with Chronic Heart Failure: Exploring Patient, Informal Caregiver, and Healthcare Professional Perceptions’, *International journal of environmental research and public health*, 17(8). doi:10.3390/ijerph17082666.

24. Shamali, M. *et al.* (2019) ‘Dyadic effects of perceived social support on family health and family functioning in patients with heart failure and their nearest relatives: Using the Actor-Partner Interdependence Mediation Model’, *PloS one*, 14(6), p. e0217970. doi:10.1371/journal.pone.0217970.

25. Uğurlu, Y.K. *et al.* (2024) ‘Compassion, stress and coping strategies in family caregivers of patients with heart failure’, *Geriatric nursing (New York, N.Y.)*, 59, pp. 357–361. doi:10.1016/j.gerinurse.2024.07.019.

26. Westland, H. *et al.* (2024) ‘Optimizing support before and after cardiac resynchronization therapy implantation in co-creation with patients with heart failure, informal caregivers, and healthcare professionals’, *European journal of cardiovascular nursing*, 23(8), pp. 886–894. doi:10.1093/eurjcn/zvae094.

27. Wu, Q. *et al.* (2024) ‘Self-care challenges of patients with heart failure from the perspectives of patients and caregivers: A qualitative study’, *Geriatric nursing (New York, N.Y.)*, 58, pp. 446–458. doi:10.1016/j.gerinurse.2024.06.005.

28. Yang, W. *et al.* (2023) ‘Advanced heart failure patients and family caregivers health and function: randomised controlled pilot trial of online dignity therapy’, *BMJ supportive & palliative care* [Preprint]. doi:10.1136/spcare-2022-003945.

29. Zhao, C. *et al.* (2024) ‘Predictors of quality of life in primary caregivers of patients with heart failure: A model of health literacy and caregiving burden’, *Heart & lung : the journal of critical care*, 65, pp. 78–83. doi:10.1016/j.hrtlng.2024.02.006.
